# Correlation of CRP/Albumin ratio and low serum albumin with the risk of major adverse cardiovascular events in elderly patients with chronic total occlusion

**DOI:** 10.1186/s41065-025-00622-1

**Published:** 2025-12-29

**Authors:** XiaoQian Che, QiuPing Chen, DongAn He, LingLong Fan

**Affiliations:** Department of Orthopaedics, Hangzhou Ninth People’s Hospital, No.98, Yilong Road, Yipeng Street, Qiantang District, Hangzhou City, 311225 Zhejiang Province China

**Keywords:** C-reactive protein/Albumin ratio, Chronic total occlusion, Major adverse cardiovascular events, Prognosis

## Abstract

**Objective:**

The C-reactive protein (CRP)/albumin (ALB) ratio (CAR), a key marker of inflammation and nutrition is closely associated with the risk of cardiovascular events. CAR’s potential to predict major adverse cardiovascular events (MACE) in elderly individuals with chronic total occlusion (CTO) has not been evaluated. Therefore, the aim of this study was to elucidate the correlation between CAR and the risk of MACE in elderly patients with CTO.

**Methods:**

This prospective cohort study consecutively included 134 patients with CTO. ALB and CRP concentrations were measured after patient admission, and CAR was calculated. Patients were followed up by telephone for a median period of 12 months and categorized into a MACE group (41 patients) and a no-MACE group (93 patients) according to the occurrence of MACE during the follow-up period. The relationship between CAR and the risk of MACE was analyzed by COX regression, and the performance of ALB, CRP, and CAR in predicting the risk of MACE was assessed using ROC curve. Kaplan-Meier survival curves were used to analyze patient survival, with subgroup analysis based on CAR.

**Results:**

The incidence of MACE in 134 CTO patients was 30.60%. ALB (HR, 0.693; 95% CI, 0.588–0.861; *P* = 0.001) and CAR (HR, 1.128; 95% CI, 1.050–1.212; *P* = 0.001) were shown to be independent predictors of MACE in patients with CTO. The AUC for CAR was 0.807, surpassing the AUCs of CRP at 0.766 and ALB at 0.700 individually. The ROC curve indicated that the optimal value to distinguish between MACE and no-MACE groups was 0.1210. Patients with CAR ≥ 0.1210 and ALB < 35.86 g/L had a significantly higher risk of MACE over 2 years (*P* < 0.001). Final subgroup analysis showed no significant interaction between CAR and each subgroup (interaction P: 0.056–0.859).

**Conclusion:**

CAR independently predicts MACE in CTO patients and provides superior prognostic value compared to using CRP or ALB alone.

## Introduction

 The incidence of coronary heart disease has been on the rise each year in recent years due to changes in lifestyle and diet, as well as the aging of society [1]. In the context of coronary artery disease, chronic total occlusion (CTO) is considered a major type of lesion. CTO lesions are characterized by coronary angiography showing thrombolysis in myocardial infarction grade 0 and occlusive coronary lesions lasting 3 months or more. Epidemiological studies indicate that about 30% of lesions found in coronary angiography are CTO [2, 3]. In the studies above, the patient population spanned all age groups, without a particular focus on elderly individuals. Due to the presence of other underlying diseases, elderly patients are more likely to have comorbidities, complicating the treatment of CTO and heightening the risk of negative prognoses [4–6]. Additionally, individuals with CTO face a much greater risk of cardiac events compared to those suffering from coronary artery disease without complete occlusion [7]. A simple and convenient approach is sought to evaluate the prognostic regression of CTO patients for better guidance in clinical diagnosis and treatment.

C-Reactive Protein (CRP) and albumin (ALB) are key indicators of inflammation and nutrition, significantly influencing the development of coronary heart disease and the evaluation of disease prognosis. CRP is an acute-phase protein that is synthesized primarily in the liver and is significantly elevated in response to stress such as infection, inflammation, and trauma. CRP serves as an independent predictor of mortality for those experiencing acute myocardial infarction, with its levels being significantly associated with cardiovascular events and overall death rates [8, 9]. ALB, as a serum protein synthesized by hepatocytes, is not only related to immune and nutritional health, but also participates in acute and chronic inflammatory processes [10]. ALB levels are negatively correlated with inflammatory response. Hypoalbuminemia is common in diseases such as heart failure, renal failure, and acute myocardial infarction [11, 12], and low ALB levels are associated with an increased risk of several cardiovascular diseases [13, 14].

CRP/ALB ratio (CAR), a novel biomarker of inflammation, has been gradually studied [15]. CAR may not only reflect the pro-inflammatory state of the organism, but also the predominance of trophic condition [16, 17]. Therefore, it may have higher sensitivity and specificity in predicting the severity and prognosis of cardiovascular diseases. Furthermore, CAR is more significantly related to the severity and prognosis of coronary heart disease compared to CRP and ALB separately [18, 19].

This study aimed to investigate the correlation between CAR and low serum ALB levels and the risk of major adverse cardiovascular events (MACE) in elderly patients with CTO, intending to provide new biomarkers and references for risk assessment and prognosis prediction of elderly CTO patients.

## Materials and methods

### Study population

A prospective collection was made of 134 elderly patients admitted to Hangzhou Ninth People’s Hospital from January 2020 to June 2022, who underwent coronary angiography and were diagnosed with CTO. CTO was defined as complete occlusion of at least one branch on coronary angiography (TIMI grade 0) with occlusion duration ≥ 3 months [20].

Inclusion criteria: [1] Patients aged ≥ 60 years; [2] patients with typical angina symptoms or objective evidence of myocardial ischemia confirmed by exercise test, ambulatory electrocardiogram, etc.; [3] patients with complete coronary occlusion (> 2.5 mm in diameter) with TIMI grade 0 on angiography; [4] patients with occlusion for more than 3 months; and [5] patients with complete coronary angiographic data.

Exclusion criteria: ① patients with recent (< 3 months) acute myocardial infarction; ② patients with other cardiomyopathies (dilated cardiomyopathy, hypertrophic cardiomyopathy, etc.) or severe valvular disease; ③ patients with previous (< 6 months) cerebrovascular accidents or history of gastrointestinal bleeding; ④ patients with severe heart failure (NYHA cardiac function classification > 2) or cardiogenic shock; ⑤ patients with malignant tumors, autoimmune diseases, coagulation disorder, other serious blood system diseases, infectious diseases, and severe hepatic and renal insufficiency.

This prospective study complied with the Declaration of Helsinki and the standards of Hangzhou Ninth People’s Hospital Ethics Committee for Research and Clinical Trials, with informed consent from the patients and their families.

### Grouping and data collection

Patients were sorted into two groups: a MACE group with 41 patients and a no-MACE group with 93 patients, depending on the occurrence of MACE during follow-up. The MACE group was defined as patients who experienced any 1 or more of all-cause death, recurrent myocardial infarction, revascularization, heart failure exacerbation, stroke, or malignant arrhythmia. The no-MACE group was defined as patients who did not experience any of these events.

Patient demographics were collected, including baseline information on age, gender, body mass index (BMI), smoking, alcohol consumption, hypertension, diabetes mellitus, previous myocardial infarction, and stroke, angiographic characteristics, and postoperative baseline medications. Diabetes mellitus was diagnosed according to American Diabetes Association criteria [21].

### PCI treatment

Decisions regarding the use of microcatheters, forward/reverse strategies, stent selection and quantity, and other invasive procedures like intravascular ultrasound, spinning, and milling during PCI were made by the surgeon. Success in CTO-PCI is achieved when the CTO is opened, the drug-eluting stent is placed effectively, and the residual stenosis at the original CTO site is under 20%, with a TIMI grade of 2 or higher after the procedure. Patients took oral antiplatelet aggregation drugs (aspirin at 100 mg orally once daily, clopidogrel at 75 mg orally once daily, or tegretol at 90 mg orally twice daily) for at least 3 days before the procedure. If the duration of oral administration was < 3 days, a loading dose of antiplatelet aggregation drugs (aspirin at 300 mg, clopidogrel at 300 mg, or tegretol at 180 mg) was given preoperatively, and atorvastatin calcium tablets at 20 mg or rosuvastatin calcium tablets at 10 mg were administered orally once daily. Patients were given dual antiplatelet therapy (aspirin at 100 mg orally once daily; clopidogrel at 75 mg orally once daily or tegretol at 90 mg orally twice daily), atorvastatin calcium tablets at 20 mg orally once daily, or resuvastatin calcium tablets at 10 mg orally once daily after PCI). According to the patients’ specific conditions and guideline recommendations, treatments involved reducing glucose and blood pressure, with additional advice on a low-salt, low-fat diet, lifestyle changes, and regular exercise. The postoperative follow-up lasted 6 months.

### Laboratory data

Prior to coronary angiography, fasting blood samples were collected from the elbow vein of each participant. Triglycerides, cholesterol, LDL, HDL, ALB, creatinine, creatine kinase-MB isoenzyme (CK-MB), and brain natriuretic peptide (BNP) were analyzed on a Cobas Integra automated analyzer (Roche Cobas Integra 400 Plus, Roche Diagnostics, USA). High-sensitivity CRP was tested using enzyme-linked immunosorbent assay (EIA-3954, DRG International Inc., Springfield Township, USA).

### Follow-up

Patients were followed up at 6-month intervals, with the follow-up concluding in June 2024. Endpoint data were obtained from medical records, clinical visits, and/or telephone interviews by trained investigators unaware of the clinical data. The occurrence of MACE was recorded, including the primary endpoint event (all-cause mortality) and secondary endpoint events (recurrent myocardial infarction, revascularization, heart failure exacerbation [new-onset heart failure or heart failure rehospitalization], stroke, and malignant arrhythmia [pacemaker/ventricular fibrillation/ventricular tachycardia]). Median follow-up was 12 months.

### Statistical analysis

Statistical analysis was conducted using SPSS 24.0 software. Measurement data were presented as x¯ ± s or M (P25, P75), while count data were shown as frequency and percentage. The independent samples t-test was used to compare measurement data following a normal distribution, whereas the independent sample χ2 test was applied for comparing count data. Statistically significant clinical indicators from the baseline data were subjected to collinearity diagnostics, and potential risk factors were identified by using univariate Cox regression analysis, and variables with a p value of less than 0.1 were included in a multivariate Cox regression analysis to identify independent risk factors for MACE. The predictive ability of CRP, ALB, and CAR for MACE was assessed using ROC analysis. The sensitivity and specificity of each indicator, and the area under the curve (AUC) were calculated. The optimal cutoff value for ALB, CRP, CAR was determined by the Youden index, allowing for the classification of ALB and CAR into high and low groups. Survival curves without adjustments were plotted using the Kaplan-Meier method, and the log-rank test was applied to compare the two groups. Subgroup analyses were also performed to investigate whether CAR had any effect on different subgroups including age, BMI, smoking history, multivessel disease, hemodialysis, LVEF%, BNP, ALB, and CRP. Differences were considered statistically significant at *P* < 0.05.

## Results

### Clinical data between MACE and no-MACE groups

A total of 134 CTO patients were enrolled, including 43 females (32.10%) and 92 males (67.90%). The median age of the patients was 65.0 (60.0, 71.0) years. The incidence of MACE after admission was 30.60%. Based on the occurrence of MACE, patients were categorized into the no-MACE group (*n* = 93) and the MACE group (*n* = 41). Patients in the MACE group were characterized by older age, increased BMI (*P* < 0.001), and reduced LVEF (%) relative to those in the no-MACE group. The number of patients with smoking history was significantly higher in the MACE group than in the no-MACE group. Multivessel disease and hemodialysis accounted for 87.80% and 51.22% in the MACE group, respectively. Patients in the MACE group had higher BNP levels (334.21 [299.71, 370.67]), CRP levels (4.82 [3.35, 5.01]), and CAR values (0.129 [0.099, 0.155]) compared with those in the no-MACE group on admission, whereas ALB levels (36.49 ± 4.64) and LVEF (%) (48.24 ± 7.36) were lower (*P* < 0.05). The other covariates did not show statistical significance between the two groups (*P* > 0.05) (Table 1).

**Table 1:** Baseline characteristics between patients in the MACE and non-MACE groups

Variables	MACE group (*n* = 41)	No-MACE group (*n* = 93)	*P* value
Age	70 (63, 73)	66 (63, 69)	0.025
Gender			
Male	29 (69.05%)	63 (67.74%)	> 0.999
Female	13 (30.95%)	30 (32.26%)	
BMI	24.87 ± 2.61	23.82 ± 2.34	0.033
Smoking history	35 (85.37%)	56 (60.22%)	0.005
Drinking history	22 (53.66%)	43 (46.24%)	0.458
Hypertension	21 (51.22%)	53 (56.99%)	0.575
Diabetes mellitus	12 (29.27%)	24 (25.81%)	0.678
Dyslipidemia	9 (21.95%)	23 (24.73%)	0.828
Previous myocardial infarction	23 (56.10%)	49 (52.69%)	0.851
Previous stroke	7 (17.07%)	11 (11.83%)	0.42
Angiographic characteristics			
Multivessel disease	36 (87.80%)	66 (69.89%)	0.047
Ostial lesion	7 (17.07%)	15 (19.35%)	0.815
Bifurcation	13 (31.71%)	33 (35.48%)	0.699
Number of occluded vessels			0.190
One	17 (41.46%)	51 (54.84%)	
Two or more	24 (58.54%)	42 (45.16%)	
Revascularization	21 (51.22%)	66 (70.97%)	0.321
Postoperative baseline medications(cases)			
Aspirin	41 (100%)	134 (100%)	1
Beta-blocker	27 (65.85%)	63 (67.74%)	0.844
Clopidogrel	38 (92.68%)	79 (84.95%)	0.27
Tegretol	8 (19.51%)	14 (15.05%)	0.614
Atorvastatin Calcium Tablets	34 (82.93%)	75 (80.65%)	0.815
Rosuvastatin Calcium Tablets	6 (14.63%)	16 (17.20%)	0.804
LVEF (%)	48.24 ± 7.36	52.79 ± 8.47	< 0.001
Laboratory tests			
TG (mmol/l)	1.36 ± 0.32	1.47 ± 0.39	0.108
TC (mmol/l)	3.76 ± 0.68	3.92 ± 0.76	0.249
LDL (mmol/l)	2.42 ± 0.48	2.28 ± 0.71	0.252
HDL (mmol/l)	0.93 ± 0.15	0.98 ± 0.19	0.138
ALB (g/l)	36.49 ± 4.64	39.63 ± 3.97	< 0.001
CK-MB (U/L)	27.63 ± 7.12	26.65 ± 7.33	0.473
BNP (pg/mL)	334.21 (299.71, 370.56)	264.76 (233.19, 317.96)	< 0.001
cTnI/(ng/mL)	0.15 ± 0.06	0.14 ± 0.05	0.318
CRP (mg/L)	4.82 (3.35, 5.01)	3.5 (2.34, 4.15)	< 0.001
CAR	0.129 (0.099, 0.155)	0.085 (0.061, 0.107)	< 0.001

### ALB and CAR are risk factors for MACE in CTO patients

The covariates with statistically significant (*P* < 0.05) differences in Table 1 were subjected to collinearity diagnostics, which showed that there was no significant multicollinearity among the variables after the exclusion of CRP, as shown in Table 2. These factors were included in the univariate COX regression analyses. Unadjusted CAR (HR, 1.188; 95% CI, 1.119–1.262; *P* < 0.001) and ALB (HR, 0.878; 95% CI, 0.817–0.944; *P* < 0.001) were significantly associated with MACE (Table 3). Covariates and potential risk factors with *P* < 0.1 in Table 3 were then included in multivariate COX regression analysis. CAR and MACE occurrence were adjusted in CTO patients using the COX proportional risk model (Table 3). In model I, CAR (HR, 1.133; 95% CI, 1.056–1.215; *P* < 0.001), and ALB (HR, 0.910; 95% CI, 0.842–0.985; *P* = 0.019) were significantly associated with the occurrence of MACE. Further, after adjusting age, gender, BMI, smoking history, LVEF%, and BNP in model II, CAR (HR, 1.100; 95% CI, 1.020–1.187; *P* = 0.014) and ALB (HR, 0.921; 95% CI, 0.847–1.002; *P* = 0.055) were also independent predictors. Model III executed a multivariate COX regression analysis without ALB and CRP, as these were strong correlates with CAR in Model II. CAR (HR, 1.128; 95% CI, 1.050–1.212; *P* = 0.001) remained an independent predictive predictor for MACE in patients with CTO (Table 4).

**Table 2:** Collinearity diagnostics

Variables	β (Standard)	t	P	Allowance	VIF
Age	0.111	1.690	0.093	*0.906*	*1.104*
BMI	0.089	1.380	0.170	*0.947*	*1.056*
Smoking history	0.140	2.175	0.032	*0.944*	*1.059*
Multivessel disease	0.071	1.066	0.288	*0.896*	*1.116*
Hemodialysis	−0.034	−0.516	0.607	*0.918*	*1.09*
LVEF%	−0.149	−2.219	0.028	*0.877*	*1.14*
BNP	0.296	4.456	< 0.001	*0.893*	*1.12*
ALB	−0.159	−2.355	0.020	*0.865*	*1.156*
CAR	0.343	4.985	< 0.001	*0.832*	*1.202*

**Table 3:** COX regression analysis of risk factors for MACE in CTO patients

Variables	Β value	SE	Waldχ2 value	HR value (95%CI)	*P* value
Age	0.074	0.034	4.658	1.077 (1.007–1.152)	0.031
BMI	0.112	0.064	3.084	1.118 (0.987–1.267)	0.079
Smoking history	0.882	0.443	3.964	2.416 (1.014–5.755)	0.046
Multivessel disease	0.845	0.483	3.059	2.329 (0.903–6.004)	0.08
Revascularization	−0.51	0.313	2.663	0.601 (0.325–1.108)	0.103
LVEF%	−0.076	0.03	6.283	0.927 (0.874–0.984)	0.012
BNP	0.012	0.003	17.483	1.012 (1.006–1.018)	< 0.001
ALB	−0.13	0.037	12.585	0.878(0.817–0.944)	< 0.001
CRP	0.458	0.099	21.234	1.581(1.301–1.920)	< 0.001
CAR	0.173	0.031	31.342	1.188 (1.119–1.262)	< 0.001

**Table 4:** Multivariate COX analysis of risk factors for MACE in CTO patients

Variables	Β value	SE	Waldχ2 value	HR value (95%CI)	*P* value
Model Ⅰ					
Age	0.067	0.039	3.037	1.070 (0.992–1.154)	0.081
Gender	0.18	0.365	0.243	1.197 (0.586–2.446)	0.622
BMI	0.125	0.066	3.511	1.133 (0.994–1.290)	0.061
ALB	−0.096	0.038	6.42	0.908 (0.843–0.978)	0.011
CAR	0.127	0.035	13.072	1.135 (1.060–1.216)	< 0.001
Model Ⅱ					
Age	0.02	0.04	0.255	1.021 (0.943–1.105)	0.614
Gender	0.056	0.367	0.023	1.058 (0.515–2.172)	0.878
BMI	0.073	0.066	1.245	1.076 (0.946–1.224)	0.265
Smoking history	0.475	0.474	1.002	1.608 (0.635–4.074)	0.317
LVEF%	−0.045	0.033	1.856	0.956 (0.896–1.020)	0.173
Multivessel disease	0.088	0.505	0.03	1.091 (0.406–2.935)	0.862
BNP	0.008	0.003	5.567	1.008 (1.001–1.014)	0.018
CRP	2.884	1.236	5.443	17.878 (1.586–201.547.586.547)	0.02
ALB	−0.389	0.147	7.05	0.678 (0.509–0.903)	0.008
CAR	−0.917	0.435	4.432	0.400 (0.170–0.939)	0.035
Model Ⅲ					
Age	0.017	0.034	0.239	1.017 (0.951–1.087)	0.625
Gender	−0.089	0.345	0.066	0.915 (0.465–1.800.465.800)	0.797
BMI	0.046	0.065	0.497	1.047 (0.922–1.188)	0.481
Smoking history	0.488	0.06	1.122	1.629 (0.661–4.016)	0.289
LVEF%	−0.03	0.032	0.891	0.970 (0.910–1.033)	0.345
Multivessel disease	0.477	0.497	0.922	1.612 (0.608–4.270)	0.337
BNP	0.005	0.003	2.562	1.005 (0.999–1.011)	0.109
CAR	0.121	0.037	10.829	1.128 (1.050–1.212)	0.001

### ALB level and CAR predict MACE occurrence in CTO patients

**Fig. 1:**
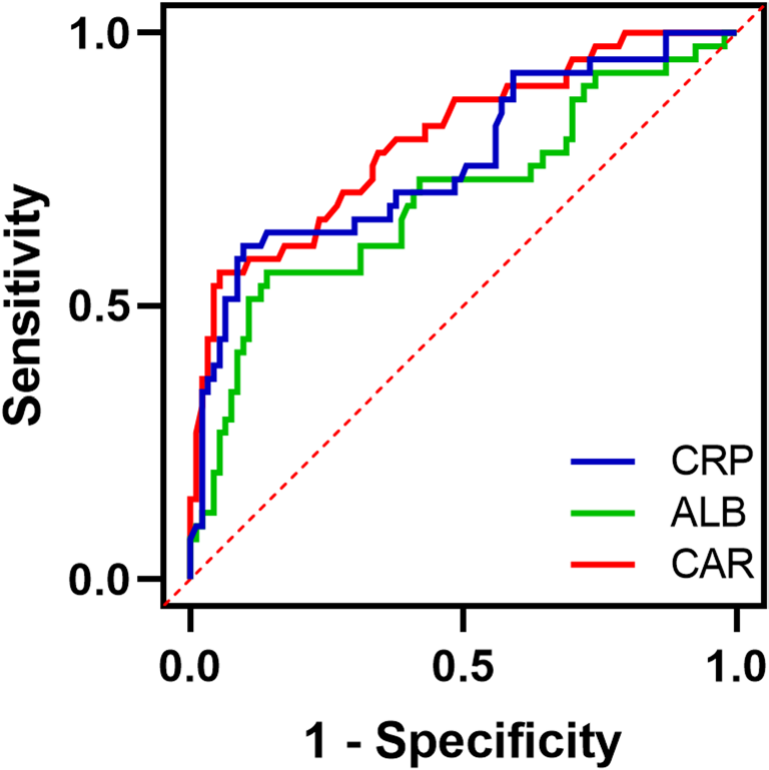
ROC curves of the predictive value of ALB, CRP, and CAR for MACE occurrence in CTO patients

**Fig. 2:**
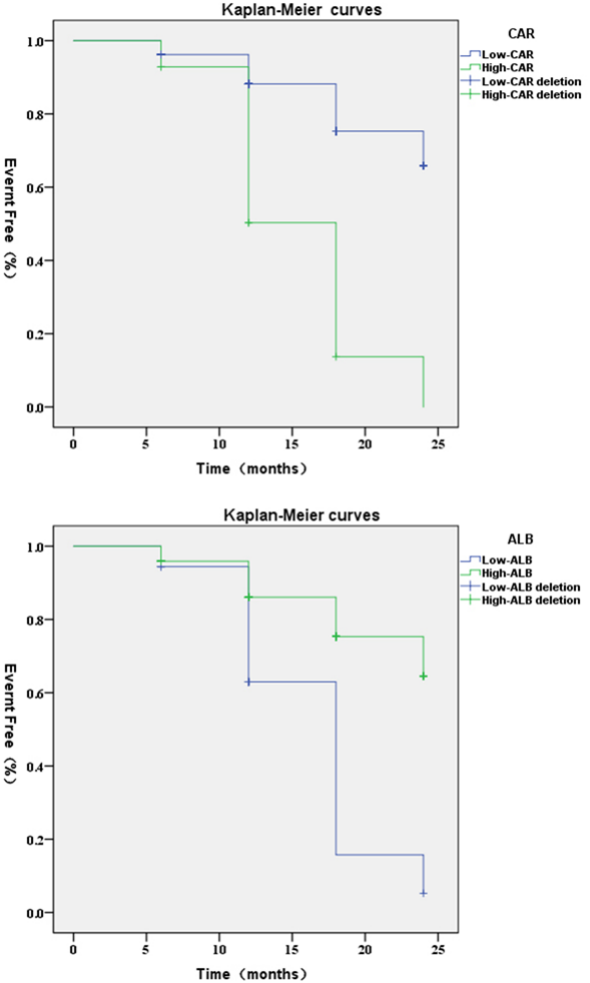
Kaplan-Meier survival analysis curves for CTO patients with different CAR and ALB levels

ROC curves for ALB, CRP, and CAR were created to predict MACE in CTO patients (Fig. 1; Table 5). The AUC of CAR (0.807 [95% CI: 0.724–0.889]) was significantly higher than that of CRP (0.766 [95% CI: 0.674–0.859]) and ALB (0.700 [95% CI: 0.595–0.801]). Thus, CAR had a significant predictive advantage. The optimal cutoff value for CAR was 0.1210, with a sensitivity of 58.54% and a specificity of 94.62%. The CTO patients were categorized into high CAR group (CAR ≥ 0.1210, *n* = 29) and low CAR group (CAR < 0.1210, *n* = 105), as well as high ALB group (ALB ≥ 35.86, *n* = 98) and low ALB group (ALB < 35.86, *n* = 36) based on the optimal cutoff value. The Kaplan-Meier survival curves (Fig. 2) found that the risk of MACE was significantly higher in patients in the high CAR and low ALB groups than in the low CAR and high ALB groups, respectively (*P* < 0.001).

**Table 5:** Predictive value of ROC curves analyzing ALB, CRP, and CAR for predicting the occurrence of MACE in CTO patients

Variables	AUC(95%CI)	Cut-off value	Specificity	Sensitivity
ALB	0.700(0.595–0.801)	35.86	86.02%	56.10%
CRP	0.766(0.674–0.859)	4.555	90.32%	60.98%
CAR	0.807(0.724–0.889)	0.121	94.62%	58.54%

### Subgroup analysis

**Fig. 3:**
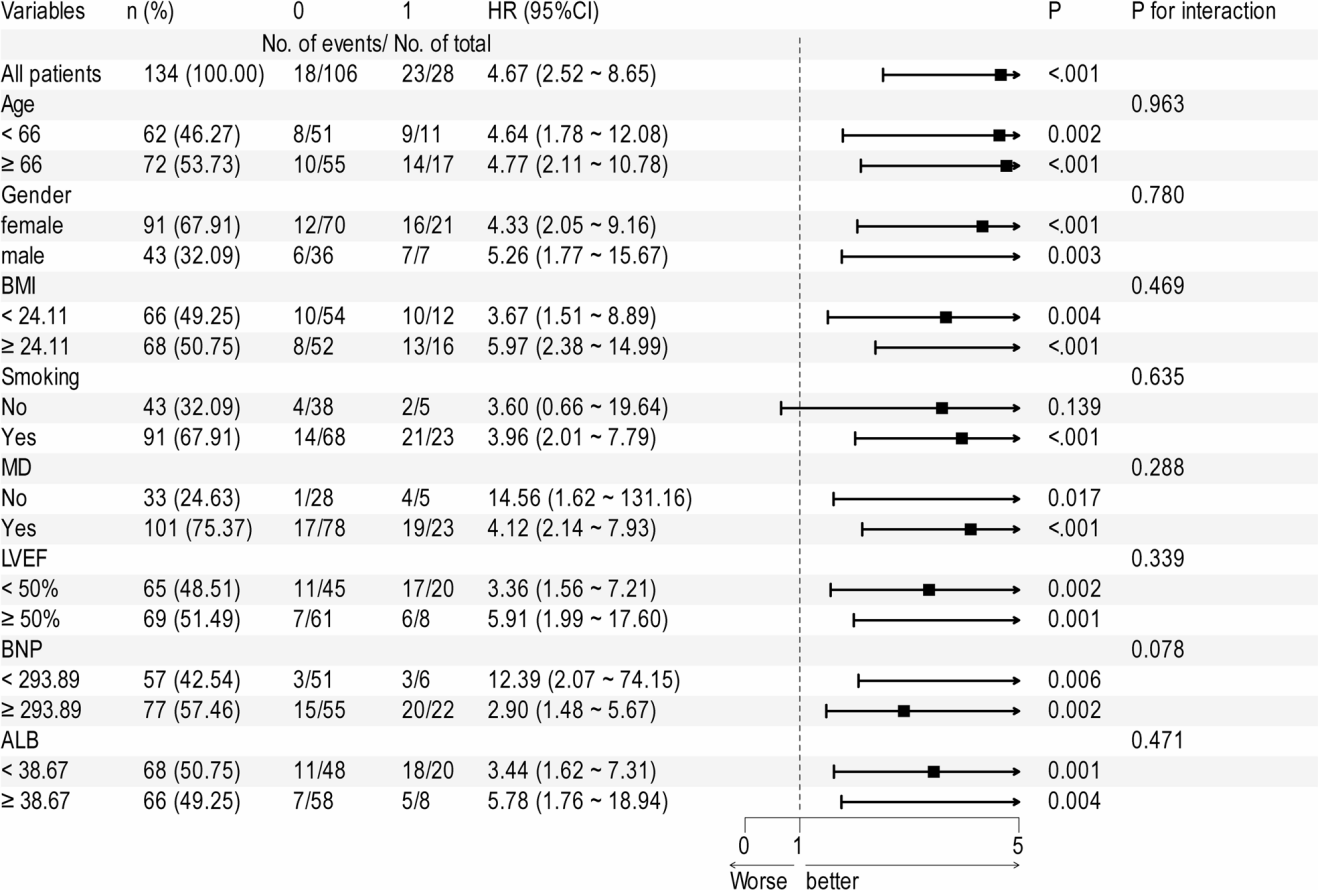
Forest plot of subgroup analysis of the relationship between MACE occurrence and CAR

Subgroup analyses were performed for all adjusted covariates. Data with normal distribution were stratified by mean: BMI, BNP, ALB; data with skewed distribution were stratified by median: age; LVEF% was stratified using the lower limit of the normal range (50%). When age, gender, BMI, smoking history, LVEF%, BNP, CRP, and ALB were stratified and analyzed, the forest plot (Fig. 3) showed no significant interaction between CAR and the subgroups (interaction P: 0.056 to 0.859).

## Discussion

Coronary artery disease, when it manifests as CTO, is highly severe, with significant morbidity and mortality rates, especially in the elderly. Inflammatory markers such as CRP, ALB, and CAR have gained importance in recent years for predicting and assessing coronary artery disease and its complications [22, 23]. However, the impact of CAR on the long-term prognosis of patients with CTO lesions has not been clarified. This study demonstrated the value of CAR in predicting MACE after PCI in patients with CTO lesions. In this prospective study, CAR was an independent factor for MACE in CTO patients. CAR (0.806) demonstrated greater accuracy than CRP (0.766) or ALB (0.700) alone, according to AUC value comparisons. Meanwhile, CTO patients with CAR > 0.1210 had a significantly higher incidence of MACE over 2 years than those with CAR < 0.1210, and subgroup analyses further supported this view.

Serum ALB constitutes the largest portion of plasma and serves as the most sensitive indicator of nutritional health. Its synthesis is adversely affected by malnutrition and chronic inflammation. ALB is a negative inflammatory protein involved in the acute inflammatory response in the body and has protective anti-inflammatory properties [24]. Studies indicate a strong association between serum ALB levels and cardiovascular disease, with low plasma ALB levels serving as an independent risk factor for cardiovascular and all-cause mortality [25, 26]. In this study, it was found that the incidence of MACE was greater in the patients with low ALB. Research indicates a significant independent link between different inflammatory indicators and coronary artery disease, with leukocytes and their variants being the predominant lab metrics for evaluating inflammation, and these metrics are highly predictive for coronary artery disease [18]. The findings also suggested that ALB holds predictive value in CTO patients, with an AUC of 0.700.

CRP synthesized by hepatocytes is used as a biomarker of inflammation and infection and a predictor of cardiovascular events [27, 28]. CRP expression is stimulated by many pro-inflammatory cytokines like IL-1β, TNF-α, and IL-6, which are known for their sensitivity as inflammation markers and are frequently monitored clinically. CRP levels quickly increase when the body becomes inflamed [29], and the predictive assessment value of CRP for cardiovascular and cerebrovascular diseases has been increasingly emphasized. In our study, patients who developed MACE had higher CRP levels. High CRP is independently associated with poor outcome and death in individuals suffering from coronary artery disease, aortic valve disease, atrial fibrillation, and congestive heart failure after coronary artery bypass grafting and heart transplantation [30–33].

CAR is an emerging marker for determining systemic inflammation, and this metric is more easily obtained from clinical biochemical tests. It can be used as an effective prognostic indicator for diseases, including coronary heart disease [34]. The composite indicator provides better value for clinical diagnosis than a single indicator by reflecting its impact on disease from multiple dimensions. When ALB decreases and CRP increases, CAR may reflect compromised immune function, significant inflammation, and a poor prognosis in patients [35, 36]. CAR is superior to CRP or ALB alone in predicting the severity and prognosis of cardiovascular disease [22]. Composite inflammatory or nutritional indicators have been found to be prognostic tools for cardiovascular disease including STEMI and chronic coronary syndromes [37]. HALP, a composite of nutritional status and systemic inflammation, is a significant independent predictor of in-hospital mortality in STEMI patients treated with PCI [38]. Consistent with these studies, our study found that elevated CAR was an independent predictor for increased risk of MACE. CAR reflects the relationship between CRP and ALB levels, highlighting both pro-inflammatory and nutritional conditions, with prognostic relevance tied to systemic inflammation [39]. CAR is associated with atherosclerosis, offering greater predictive power for coronary heart disease prognosis than either CRP or ALB alone [19, 40, 41]. Similarly, our study showed that CAR had an AUC value of 0.807 in predicting the risk of MACE in CTO patients, which was superior to CRP and ALB alone. Finally, this study only compared the predictive performance of CAR with that of single indicators through AUC analysis, lacking validation using the Net Reclassification Index (NRI) and Integrated Discrimination Improvement (IDI). Consequently, it exhibits certain limitations in the depth and comprehensiveness of model validation. Future research may incorporate these advanced model validation techniques to more scientifically and accurately assess the predictive performance of indicators such as CAR.

There are some limitations in this study that warrant attention. First, this study is a single-center prospective cohort study with a relatively small sample size of 134 patients. All subjects were recruited from the same hospital, potentially introducing selection bias and limiting the generalizability of the findings. Future studies should be conducted as multicenter, large-sample prospective investigations, incorporating CTO patients from diverse geographic regions and hospitals to further validate the predictive value of CAR and enhance the generalizability of the findings. Second, only the CAR value at admission was measured, without assessing dynamic changes during medication. This study included only a median follow-up of 12 months, leaving the long-term prognosis unclear. Extending the follow-up period to observe the relationship between CAR and the long-term risk of MACE in CTO patients would facilitate a more comprehensive evaluation of CAR’s predictive value. Third, CRP, ALB, and CAR were evaluated as prognostic biomarkers for CTO patients, but data on other inflammatory markers were lacking. Furthermore, the study only included CTO patients who underwent PCI, suggesting that the findings may not be generalizable to all CTO patients. Finally, relying solely on AUC comparisons to evaluate the predictive performance of CAR versus single indicators, without validation through the NRI and IDI, results in certain limitations in the depth and comprehensiveness of model validation. Future research should incorporate these advanced model validation techniques to more scientifically and accurately assess the predictive performance of metrics such as CAR.

In conclusion, CAR and low serum ALB levels are closely associated with the risk of MACE in elderly patients with CTO. Higher CAR combined with lower ALB points to inflammation and oxidative stress, independently predicting MACE. In clinical practice, prioritizing the detection and assessment of ALB and CAR is essential for the early identification of high-risk patients and the adoption of proactive and effective strategies. For high-risk patients with high CAR levels, it may be useful to consider more aggressive interventions, such as targeted improvement of nutritional status (correcting low ALB-related nutritional deficiencies), moderate control of chronic inflammation (e.g., optimizing management of the underlying disease to reduce inflammation), and intensified secondary prevention therapies, such as antiplatelet and lipid modulation, to reduce the risk of MACE. Further research is required to investigate the precise mechanisms of CAR and low serum ALB levels in elderly CTO patients, as well as ways to enhance patient outcomes by modifying these indicators.

## Data Availability

Data is available from the corresponding author on request.
